# Fuzzy Kolmogorov Complexity Based on a Classical Description

**DOI:** 10.3390/e22010066

**Published:** 2020-01-04

**Authors:** Songsong Dai

**Affiliations:** School of Electronics and Information Engineering, Taizhou University, Taizhou 318000, China; ssdai@tzc.edu.cn

**Keywords:** fuzzy Turing machines, fuzzy languages, Kolmogorov complexity

## Abstract

In this paper, we give a definition for fuzzy Kolmogorov complexity. In the classical setting, the Kolmogorov complexity of a single finite string is the length of the shortest program that produces this string. We define the fuzzy Kolmogorov complexity as the minimum classical description length of a finite-valued fuzzy language through a universal finite-valued fuzzy Turing machine that produces the desired fuzzy language. The classical Kolmogorov complexity is extended to the fuzzy domain retaining classical descriptions. We show that our definition is robust, that is to say, the complexity of a finite-valued fuzzy language does not depend on the underlying finite-valued fuzzy Turing machine.

## 1. Introduction

Kolmogorov–Solomonoff–Chaitin (Kolmogorov, for short) complexity was introduced independently by Solomonoff [1] and Kolmogorov [2] and later by Chaitin [3]. It is based on the discovery of the universal Turing machine that can simulate any other Turing machine [4,5]. Kolmogorov complexity of a single finite string is the length of the shortest program for a universal Turing machine that correctly produces this string as its output. It also is a measure of the amount of information contained in the string. It has been proved that, although there are many Turing machines, the length of shortest programs are invariant, which can differ by at most an additive constant, under the choice of the underlying Turing machine [6]. The theory of Kolmogorov complexity has been widely used on areas such as question answering system [7], combinatorics [8], learning theory [9], bioinformatics [10] and cryptography [11,12].

In 1985, Deutsch [13] introduced quantum Turing machines as theoretical models of quantum computers. Quantum Turing machines expand the classical model of Turing machines since they allow quantum interference to take place on their computation paths. Bernstein and Vazirani [14] showed that a quantum Turing machine can be universal in the approximate sense. Recently, some quantum versions of Kolmogorov complexity were proposed by several researchers. Vitányi [15] proposed a definition for quantum Kolmogorov complexity, which measures the amount of classical information necessary to approximate the quantum state. Berthiaume et al. [16] proposed a new definition of quantum Kolmogorov complexity of a quantum bit string by the length of the shortest quantum input for a universal quantum computer that outputs the desired string.

The first formulation of fuzzy computation was introduced by Zadeh [17], who defined the notion of fuzzy algorithms based on a fuzzification of Turing machines and Markov algorithms. Then, Lee and Zadeh [18] defined the concept of fuzzy languages. Santos [19] demonstrated the equivalence between fuzzy algorithms and fuzzy Turing machines. Next, Wiedermann [20] considered the computability and complexity of fuzzy computation. With Wiedermann’s work, Bedregal and Figueira [21] proved that there is no universal fuzzy Turing machine that can simulate every fuzzy Turing machine. Afterward, Li [22,23] studies some variants of fuzzy Turing machines. He showed that a universal fuzzy Turing machine exists in some approximate sense and also showed that a universal fuzzy Turing machine exists in the frame of finite-valued logic. Li [24] subsequently studied the lattice-valued fuzzy Turing machines where the lattice is a distributive lattice. In [24], He showed that a universal lattice-valued fuzzy Turing machine exists if the distributive lattice is finite. Li et al. [25] also showed that a universal orthomodular lattice-valued fuzzy Turing machine exists if the orthomodular lattice is finite. More recently, Wu et al. [26] established a theory of Turing machines based on complete residuated lattice-valued logic and also showed that a universal machine exists if the lattice is a finite complete residuated lattice with universal bounds 0 and 1.

With the development of fuzzy computation, it is natural to ask how to define the Kolmogorov complexity of fuzzy languages (or fuzzy sets). What are the problems when developing a theory of fuzzy Kolmogorov complexity?

One problem is: which model of fuzzy computation? They are many formal models of fuzzy computation such as fuzzy P systems, fuzzy labeled transition systems, and fuzzy Turing machines. Similar to traditional Kolmogorov complexity, the fuzzy Turing machine model is the appropriate one to consider. To define fuzzy Kolmogorov complexity by way of fuzzy Turing machines causes two problems:

- There are continuously many fuzzy languages.
- There are continuously many fuzzy Turing machines.

We can finitely describe only a countable subset and a universal fuzzy Turing machine only exists in the the frame of finite-valued logic, i.e., the truth values of membership degrees of fuzzy languages are finite. So, in this paper, we consider to define the theory of fuzzy Kolmogorov complexity of finite-valued fuzzy languages based on finite-valued fuzzy Turing machines.

One next problem is: what does “length” mean? In the classical setting, the amount of information of a string is measured in bits. How to measure the amount of information of a fuzzy language? For a fuzzy language, the size of its support is not an appropriate measure since it ignores the truth value or membership degree of a string and the length differences between strings. Since the set of our objects (finite-valued fuzzy languages) is countable. Hence, we consider classical descriptions of fuzzy languages, i.e., measure the amount of information about fuzzy languages in bits.

The rest of this paper is organized as follows: In Section 2, we give basic notation that will be used in the paper, and recall the definitions and some properties of finite-valued fuzzy Turing machines and classical Kolmogorov complexity. In Section 3, we give our definition of fuzzy Kolmogorov complexity and show the invariance theorem for this definition. Some properties of fuzzy Kolmogorov complexity are given in Section 4. Some discussions on research of fuzzy Kolmogorov complexity are made in Section 5. Conclusions are briefly summed up in Section 6.

## 2. Preliminaries

### 2.1. Notation

Let Σ={0,1}, the set of all finite binary strings is denoted by $\Sigma^{*}$(or ${\left\{0,1\right\}}^{*}$), and the set of length *m* strings is denoted by $\Sigma^{m}$(or ${\left\{0,1\right\}}^{m}$). Then, l(x) denotes the length of the string *x* in bits. For the cardinality of a set *A* we write |A|. In general, we use x,y,z,⋯ to denote finite binary strings. ϵ denotes the empty string.

A language is just a set of finite strings, a fuzzy language can be defined as a fuzzy subset of finite strings. In this paper, we only consider the fuzzy languages in the the frame of finite-valued logic, i.e, the membership degrees of fuzzy languages are finite. Let $D_{n}=\left\{0,a_{1},a_{2},\cdots ,a_{n-2},1\right\}$. A *n*-valued fuzzy language is a map $L:\Sigma^{*}\to D_{n}$. It is a *n*-valued fuzzy subset of $\Sigma^{*}$. We denote by $L_{n}\left(\Sigma^{*}\right)$ the set of *n*-valued fuzzy languages over $\Sigma^{*}$, and denote by $L_{n}\left(\Sigma^{m}\right)$ the set of *n*-valued fuzzy languages over $\Sigma^{m}$.

Fuzzy languages also mean possibility distributions over $\Sigma^{*}$. For any $L\in L_{n}\left(\Sigma^{*}\right)$, its support, Supp(L), can be represented by $\left\{x|x\in \Sigma^{*}\wedge L\left(x\right)\neq 0\right\}$. If the support of a *n*-valued fuzzy language is the set $\left\{x_{1},x_{2},\ldots ,x_{m}\right\}\subseteq \Sigma^{*}$, we may write *L* in Zadeh’s notation [27] as

$$L=\frac{L(x_{1})}{x_{1}}+\frac{L(x_{2})}{x_{2}}+\cdots +\frac{L(x_{m})}{x_{m}},$$

where $\frac{L(x_{i})}{x_{i}}$ means that $L(x_{i})$ is the truth value or membership grade of $x_{i}$ in *L*.

### 2.2. Finite-Valued Fuzzy Turing Machines

Our model of computation is finite-valued fuzzy Turing machines with three tapes: a one-way infinite classical input (the program) tape, a one-way infinite auxiliary (conditional) tape containing finite-valued fuzzy languages, and a one-way infinite output tape containing finite-valued fuzzy languages. A finite-valued fuzzy Turing machine *U* with classical program *p* and auxiliary (classical or fuzzy) input *L* computes until it halts with output U(p,L) on its output tape.

**Definition** **1.** *Define the output U(p,x) of a finite-valued fuzzy Turing machine U with classical program p and auxiliary (classical or fuzzy) input x as the finite-valued fuzzy language L resulting from U computing until it halts with output L on its output tape, denoted U(p,x)=L. When the auxiliary input is a fuzzy language $L^{\prime }$, we can write $U(p,L^{\prime })=L$. When the auxiliary input is the empty string ϵ, we simply write U(p) instead of U(p,ϵ).*

For simplicity, all finite-valued fuzzy Turing machines considered in this paper takes values in $D_{n}$. As mentioned in [23,24,25,26], universal fuzzy Turing machine exists in the frame of finite-valued logic. There are countably many *n*-valued fuzzy Turing machine, we can assign Gödel numbers to each *n*-valued fuzzy Turing machine, and obtain $M_{1},M_{2},\cdots$ an enumeration of *n*-valued Turing machines. By [23,24,25,26], there is a universal *n*-valued fuzzy Turing machine *U* simulates the others exactly

$$U\left((i_{M},p),x\right)=M_{i}\left(p,x\right)$$

for all $i_{M},p,x$, where $i_{M}$ is the index of *M* in this enumeration.

### 2.3. Classical Kolmogorov Complexity

Some relevant definitions and properties of classical Kolmogorov complexity are recalled below. For more details and attributions we refer to [6].

**Definition** **2.** (Classical Kolmogorov complexity). Let T be a fixed Turing machine. For any pair of strings $x,y\in {\left\{0,1\right\}}^{*}$, the conditional Kolmogorov complexity $K_{T}\left(y|x\right)$ of y given x (with respect to T) is defined by

$$K_{T}\left(y|x\right)=\min\left\{l(p):T(p,x)=y\right\}.$$

When *x* is the empty string ϵ, we simply write $K_{T}\left(y\right)$ instead of $K_{T}\left(y|\epsilon \right)$.

**Theorem** **1.** *(Classical invariance theorem). There is a universal Turing machine T such that for any other Turing machine $T^{\prime }$ there is a constant $c_{T^{\prime }}$ with*

$$K_{T}\left(y|x\right)\leq K_{T^{\prime }}\left(y|x\right)+c_{T^{\prime }}$$

*for all strings x and y, where the constant $c_{T^{\prime }}$ depends on $T^{\prime }$ but not on strings x and y.*

This result shows that the length of the shortest programs does not depend on more than an additive constant on the underlying Turing machine. So this definition of Kolmogorov complexity is robust.

A crucial fact of Kolmogorov complexity is the existence of incompressible strings, which is the so-called incompressibility theorem. It is a simple but powerful tool used frequently in the theory and application of classical Kolmogorov complexity. It can be stated as follows.

**Theorem** **2.** *(Classical incompressibility). For any length m, there is a length m string x such that K(x)≥m.*

Its proof is based on the pigeonhole principle. For each *m*, there are $2^{m}$ binary strings of length *m*, but the number of programs of length smaller than *m* is $2^{m}-1$ in total. Therefore, there is at least one length *m* string *x* with K(x)≥m.

## 3. Classical Descriptions of Fuzzy Languages

In this section, we define the fuzzy Kolmogorov complexity of fuzzy languages and show that our definition is robust.

### 3.1. Definitions

Suppose that all finite-valued fuzzy languages and finite-valued fuzzy Turing machines considered in this paper taking values in $D_{n}$, denoted *n*-valued fuzzy languages and *n*-valued fuzzy Turing machines respectively. We can naturally define the fuzzy Kolmogorov complexity of a *n*-valued fuzzy language (with respect to a *n*-valued fuzzy Turing machine) as follows.

**Definition** **3.** *(Fuzzy Kolmogorov complexity). For any n-valued fuzzy Turing machine U and pair of n-valued fuzzy languages $L,L^{\prime }$, the conditional fuzzy Kolmogorov complexity $FK_{U}\left(L|L^{\prime }\right)$ of the fuzzy language L with respect to U with given auxiliary (conditional) input $L^{\prime }$ is the length of the smallest bit string p such that $U(p,L^{\prime })=L$, i.e.,*

$$FK_{U}\left(L|L^{\prime }\right)=\min\left\{l\left(p\right):U\left(p,L^{\prime }\right)=L\right\}.$$

*The (unconditional) fuzzy Kolmogorov complexity $FK_{U}\left(L\right)$ of L is defined as $FK_{U}\left(L|\epsilon \right)$, if auxiliary (conditional) input $L^{\prime }$ is the empty string.*

Note that a program is a classical input for a fuzzy Turing machine in our definition. l(p) is the number of bits in the program *p*. Therefore, we measure the amount of information of a fuzzy language in bits. This definition gives a classical description of finite-valued fuzzy languages through universal finite-valued fuzzy Turing machines.

### 3.2. Invariance

To show that our definition is robust we must show that the complexity of a *n*-valued fuzzy language does not depend on the underlying *n*-valued fuzzy Turing machine.

We use the existence of a universal *n*-valued fuzzy Turing machine to prove the invariance theorem for our definition.

**Theorem** **3.** *(Invariance theorem). There is a universal n-valued fuzzy Turing machine U such that for any n-valued fuzzy Turing machine M and n-valued fuzzy language L*

$$FK_{U}\left(L\right)\leq FK_{M}\left(L\right)+c_{M},$$

*where $c_{M}$ is a constant depending only on M.*

**Proof.**  The proof of this theorem follows from the existence of a universal *n*-valued fuzzy Turing machine, as proven in [23,24,25]. We fix $M_{1},M_{2},\cdots$ as a standard enumeration of *n*-valued fuzzy Turing machines using Gödel numbers. Let *U* be a universal *n*-valued fuzzy Turing machine in this enumeration that simulates the others exactly. Let *p* be the program that witnesses that $FK_{M}\left(L\right)=l\left(p\right)$. *U* can simulate the behavior of *M*, $U\left(i_{M},p\right)=M\left(p\right)$, where $i_{M}$ is the index of *M* in this enumeration. Let *q* be the program that witnesses that $FK_{U}\left(L\right)=l\left(q\right)$. By the definition of fuzzy Kolmogorov complexity, $l\left(q\right)\leq l\left(p\right)+c_{M},$ where $c_{M}$ is the length of the description of the index of *M* in the enumeration. This completes the proof of the invariance theorem. □

Moreover, we have the following result.

**Theorem** **4.** *For every pair of universal n-valued fuzzy Turing machines $U,U^{\prime }$, there is a fixed constant $c_{U,U^{\prime }}$ such that for any n-valued fuzzy language L, we have*

$$|FK_{U}\left(L\right)-FK_{U^{\prime }}\left(L\right)|\leq c_{U,U^{\prime }},$$

*where $c_{U,U^{\prime }}$ is a constant depending only on U and $U^{\prime }$.*

**Proof.**  To prove this, substitute $U^{\prime }$ for *M* in the Theorem 3, we have $FK_{U}\left(L\right)\leq FK_{U^{\prime }}\left(L\right)+c_{U^{\prime }}$, where $c_{U^{\prime }}$ is a constant depending only on $U^{\prime }$. Conversely, substitute $U^{\prime }$ for *U* and *U* for *M* in the Theorem 3, we have $FK_{U^{\prime }}\left(L\right)\leq FK_{U}\left(L\right)+c_{U}$, where $c_{U}$ is a constant depending only on *U*. Let $c_{U,U^{\prime }}=\max\left\{c_{U^{\prime }},c_{U}\right\}$, and combine the two resulting inequalities, we have $|FK_{U}\left(L\right)-FK_{U^{\prime }}\left(L\right)|\leq c_{U,U^{\prime }},$ where $c_{U,U^{\prime }}$ is a constant depending only on *U* and $U^{\prime }$. □

The same holds true for the conditional fuzzy Kolmogorov complexity of fuzzy languages.

**Theorem** **5.** *There exists a universal n-valued fuzzy Turing machine U such that for all n-valued fuzzy Turing machines M and n-valued fuzzy languages $L_{1},L_{2}$ we have*

$$FK_{U}\left(L_{1}|L_{2}\right)\leq FK_{M}\left(L_{1}|L_{2}\right)+c_{M},$$

*where $c_{M}$ is a constant depending only on M.*

**Theorem** **6.** *For every pair of universal n-valued fuzzy Turing machines $U,U^{\prime }$, there is a fixed constant $c_{U,U^{\prime }}$ such that for any pair of n-valued fuzzy languages $L_{1},L_{2}$, we have*

$$|FK_{U}\left(L_{1}|L_{2}\right)-FK_{U^{\prime }}\left(L_{1}|L_{2}\right)|\leq c_{U,U^{\prime }},$$

*where $c_{U,U^{\prime }}$ is a constant depending only on U and $U^{\prime }$.*

Our results are based on the existence of a universal finite-valued fuzzy Turing machine. Since the existence of universal finite-valued fuzzy Turing machine in Ref. [23,24,25,26], the truth value domain $D_{n}$ could be fixed as a complete residuated lattice, a distributive lattice or a orthomodular lattice. Here, we do not discuss the fuzzy Kolmogorov complexity in these logic frames further.

We have shown that our definition is independent of the particular choice of the *n*-valued fuzzy Turing machine. That is, changing the fuzzy Turing machine will change the complexity at most by an additive constant.

Henceforth, we will fix a universal *n*-valued fuzzy Turing machine *U* and call *U* the reference machine. Then we simply write FK(L) instead of $FK_{U}\left(L\right)$, and $FK(L_{1}|L_{2})$ instead of $FK_{U}\left(L_{1}|L_{2}\right)$.

Based on the Invariance theorems, we have the following two results.

First, we have the following relationship between conditional fuzzy Kolmogorov complexity and (unconditional) fuzzy Kolmogorov complexity.

**Theorem** **7.** *There is a constant c, such that for each pair of n-valued fuzzy languages $L_{1},L_{2}$,*

$$FK\left(L_{1}|L_{2}\right)\leq FK\left(L_{1}\right)+c.$$

**Proof.**  To prove this inequality, construct a *n*-valued fuzzy Turing machine $U^{\prime }$ that for all $L_{2},L_{3}$ computes output $L_{1}$ on input $\left(L_{2},L_{3}\right)$ if and only if the universal ref *n*-valued fuzzy Turing machine *U* computes output $L_{1}$ on input $\left(L_{2},\epsilon \right)$. Then $KF_{U^{\prime }}\left(L_{1}|L_{2}\right)=KF_{U}\left(L_{1}\right)$. By the Theorem 5, there is a constant *c* such that $KF_{U}\left(L_{1}|L_{2}\right)\leq KF_{U^{\prime }}\left(L_{1}|L_{2}\right)+c=KF_{U}\left(L_{1}\right)+c$. Thus, we have $FK\left(L_{1}|L_{2}\right)\leq FK\left(L_{1}\right)+c.$ □

Second, we have the following relationship between classical Kolmogorov complexity and fuzzy Kolmogorov complexity for a classical string.

**Theorem** **8.** *There is a constant c, such that for every finite, classical string x, it holds that FK(x)≤K(x)+c. The constant c depends only on the underlying universal Turing machine.*

**Proof.**  This is clear: the universal *n*-valued fuzzy Turing machine can also simulate any classical Turing machine. □

## 4. Properties of Fuzzy Kolmogorov Complexity

In this section, we introduce some properties of the fuzzy Kolmogorov complexity. We find that many of the properties of the classical Kolmogorov complexity, or natural analogs thereof, also hold for the fuzzy Kolmogorov complexity.

### 4.1. Incompressibility

First, we are going to prove a fuzzy analog of the classical incompressibility theorem. It is a fuzzification of a classical incompressibility.

**Theorem** **9.** *(Incompressibility). Let $x\in {\left\{0,1\right\}}^{M}$ and $n=2^{N}$. There is an incompressible n-valued fuzzy string $\frac{L(x)}{x}$, i.e., $FK\left(\frac{L(x)}{x}\right)\geq M+N$.*

**Proof.**  The number of programs of length less than M+N is $\sum_{i=0}^{M+N-1}=2^{M+N}-1$ in total. But there are $2^{M+N}$ such *n*-fuzzy strings. Which is impossible. Hence, there is at least one *n*-valued fuzzy string such that $FK\left(\frac{L(x)}{x}\right)\geq M+N$. This proves the theorem. □

By a simple counting argument there exist fuzzy languages that are incompressible. Then, how many fuzzy languages are incompressible? It depends on both the length of strings and the domain of truth values, which can be obtained by the same counting argument as follows.

**Theorem** **10.** *Let $x\in {\left\{0,1\right\}}^{M}$ and $n=2^{N}$. At least $2^{M+N}\left(1-2^{c}\right)$n-fuzzy strings have complexity at least M+N−c.*

**Proof.**  Similarly to Theorem 9. □

We fix the L(x)=1 for the classical setting. Then we only consider the string *x* itself and can obtain the classical incompressibility theorem (Theorem 2) in a similar way.

In above Theorems, we only consider the single *n*-fuzzy string, i.e., the language $L\in L_{n}\left(\Sigma^{M}\right)$ with |Supp(L)|=1 or L=ϵ. In a similar way, we have the following results for *n*-fuzzy languages.

**Theorem** **11.** *Let $x\in {\left\{0,1\right\}}^{M}$ and $n=2^{N}$. There is a n-valued fuzzy language $L\in L_{n}\left({\left\{0,1\right\}}^{M}\right)$ such that FK(L)≥M·N.*

**Proof.**  The number of programs of length less than M·N is $\sum_{i=0}^{M\cdot N-1}=2^{M\cdot N}-1$ in total. But there are $2^{M\cdot N}$ such *n*-fuzzy languages. Which is impossible. Hence, there is at least one *n*-valued fuzzy language such that FK(L)≥M·N. This proves the theorem. □

**Theorem** **12.** *Let $x\in {\left\{0,1\right\}}^{M}$ and $n=2^{N}$. At least $2^{M\cdot N}\left(1-2^{c}\right)$n-fuzzy languages of the set $L_{n}\left({\left\{0,1\right\}}^{M}\right)$ have complexity at least M·N−c.*

**Proof.**  Similarly to Theorem 11. □

### 4.2. Subadditivity

Now we consider the subadditivity property of fuzzy Kolmogorov complexity. It can be stated as follows.

**Theorem** **13.** Let $\Sigma_{1},\Sigma_{2}\subseteq {\left\{0,1\right\}}^{*}$ and $\Sigma_{1}\cap \Sigma_{2}=\emptyset$, $L_{1}\in L_{n}\left(\Sigma_{1}\right)$ and $L_{2}\in L_{n}\left(\Sigma_{2}\right)$. There is constants c and $c^{\prime }$ such that

$$\begin{array}{c} FK\left(L_{1}\right)\leq FK\left(L_{1},L_{2}\right)+c \end{array}$$

*and*

$$\begin{array}{cc} FK(L_{1},L_{2})\leq & FK\left(L_{1}\right)+FK\left(L_{2}\right)\;+2\log_{2}\min\left(FK\left(L_{1}\right),FK\left(L_{2}\right)\right)+c^{\prime }. \end{array}$$

**Proof.**  The first inequality is obvious. To prove the second inequality. Suppose *p* is a shortest program to produce $L_{1}$, and *q* is a shortest program to produce $L_{2}$. Then there is a constant $c^{\prime }$ for some machine *U* to schedule the two programs. However, any such *U* will have to know where to divide its input to identify *p* and *q*. We can use input $p^{\prime }pq$ or $q^{\prime }qp$, where $p^{\prime }$ is the description of *p* with length l(p) and $q^{\prime }$ is the description of *q* with length l(q). In this way, we have $FK\left(L_{1},L_{2}\right)\leq FK\left(L_{1}\right)+FK\left(L_{2}\right)+2\log_{2}\min\left(FK\left(L_{1}\right),FK\left(L_{2}\right)\right)+c^{\prime }$ for some constant *c*. □

**Remark** **1.** *From above theorem, the inequality $FK\left(L_{1},L_{2}\right)\leq FK\left(L_{1}\right)+FK\left(L_{2}\right)$ hold within a logarithmic term. We cannot eliminate this term for the general case, since we do not define the prefix-free complexity of fuzzy languages, i.e., no program is a proper prefix of another program.*

**Theorem** **14.** Let $\Sigma_{1},\Sigma_{2}\subseteq {\left\{0,1\right\}}^{*}$ and $\Sigma_{1}\cap \Sigma_{2}=\emptyset$, $L_{1}\in L_{n}\left(\Sigma_{1}\right)$ and $L_{2}\in L_{n}\left(\Sigma_{2}\right)$. We have

$$FK\left(L_{1},L_{2}\right)\leq FK\left(L_{1}\right)+FK\left(L_{2}|L_{1}\right)+O\left(\log\left(FK(L_{1})\right)\right).$$

**Proof.**  To prove this inequality. Suppose *p* is a shortest program to produce $L_{1}$, and *q* is a shortest program to produce $L_{2}$ from $L_{1}$. Similar to the above theorem, we have to identify *p* and *q*. Hence, we can obtain this inequality. □

**Remark** **2.** *This is different from quantum Kolmogorov complexity in [16] since the no-cloning theorem. In the quantum case, producing a quantum state from another quantum state may destroy the original quantum state.*

We have the following corollary for the classical case.

**Corollary** **1.** *For each pair of classical strings x,y, we have*

$$K\left(x,y\right)\leq K\left(x\right)+K\left(y|x\right)+O\left(\log(K(x))\right).$$

This corollary is the property of classical Kolmogorov complexity in [6].

### 4.3. Upper Bound on Complexity

In the classical case, we have the following upper bound of classical Kolmogorov complexity.

**Theorem** **15.** *For each classical strings x, there is a constant c such that*

K(x)≤l(x)+c.

For the definition of fuzzy Kolmogorov complexity to be useful, the complexity of a fuzzy string should have an upper bound. Similar to the results in Incompressibility (Section 4.1), such upper bound depends on both the length of the string and the domain of truth values, which can be obtained as follows.

**Theorem** **16.** *Let $x\in {\left\{0,1\right\}}^{M}$ and $n=2^{N}$. There exists a constant c such that for each n-valued fuzzy string, $FK\left(\frac{L(x)}{x}\right)\leq M+N+2\log_{2}\min\left(M,N\right)+c.$*

**Proof.**  Suppose *p* is a shortest program to produce L(x), *q* is a shortest program to produce *x*, and a shortest program *r* to identify *p* and *q*. Since $x\in {\left\{0,1\right\}}^{M}$ and $n=2^{N}$, using Theorem 15, we have $l\left(p\right)\leq M+c^{\prime }$ and $l\left(q\right)\leq N+c^{\prime \prime }$. Using Theorem 13, we obtain this inequality. □

## 5. Discussion

Many classical quantities, e.g the Shannon entropy, have been successfully generalized to quantum information and have become useful and powerful tools to understand and further develop quantum information theory. In the case of Kolmogorov complexity, though, the way to do so is not straightforward. There are many authors doing some work on the fuzzy Turing machines, this paper uses fuzzy Turing machine to describe the complexity of fuzzy languages. Of course, many works should be done on the theory of fuzzy Kolmogorov complexity. Some of the research questions are the following.

(1) How to define the complexity of infinite-valued fuzzy languages? A good definition of fuzzy Kolmogorov complexity should be robust, i.e., invariant under the choice of the underlying fuzzy Turing machine. Our invariance theorem (Theorem 3) is proved based on the existence of a universal fuzzy Turing machine in the frame of finite-valued logic. However, a universal infinite-valued fuzzy Turing machine exists in the approximate sense. Some methods of quantum Kolmogorov complexity in [15,16] may provide some referential suggestions.
(2) What is the difference between fuzzy versions of Kolmogorov complexity in different logics (or variants of fuzzy Turing machines)? There are several types of fuzzy logics: ukasiewicz logic, Gödel logic, product logic and so on [28]. Different types of fuzzy logic have different axioms and rules. Are there different variants of fuzzy Kolmogorov complexity based on different choices of logic systems?
(3) What is the relation between fuzzy Kolmogorov complexity and fuzzy information theory? The expected value of classical Kolmogorov complexity equals the Shannon entropy (up to a constant) for any recursive probability distribution [6]. Many classical information measures have been successfully generalized to fuzzy information theory, such as non-probabilistic entropy [29,30], hybrid entropy [31], Yager Entropy [32,33] and Kolmogorov–Sinai Entropy [34,35]. They have become useful tools in theoretical and applied research of fuzzy information theory. What is the relationship between fuzzy Kolmogorov complexity and these entropy measures?
(4) What is the relation between fuzzy Kolmogorov complexity and fuzzy computational complexity? In this paper, the computation time of the fuzzy Turing machine is not limited. It is interesting to develop a time-bounded version of fuzzy Kolmogorov complexity and consider its relationship to fuzzy computational complexity theory [24].

## 6. Conclusions

In this research, we proposed a fuzzy version of Kolmogorov complexity, which defined as the minimum classical description length of a finite-valued fuzzy language through a finite-valued fuzzy Turing machine. We showed the invariance theorem for our definition. Several properties of classical Kolmogorov complexity are generalized to the case of fuzzy Kolmogorov complexity.
